# Multimodality system of x-ray and fluorescence based on Fourier single-pixel imaging for small animals

**DOI:** 10.1117/1.JBO.27.9.090501

**Published:** 2022-09-16

**Authors:** Zhuoyao Huang, Jie Zhang, Hui Gong, Xiaoquan Yang

**Affiliations:** a Huazhong University of Science and Technology, Britton Chance Center for Biomedical Photonics, Wuhan National Laboratory for Optoelectronics, MoE Key Laboratory for Biomedical Photonics, Wuhan, China; b HUST-Suzhou Institute for Brainsmatics, JITRI, Suzhou, China

**Keywords:** *in-vivo* multi-modality imaging, Fourier single-pixel imaging, small animals

## Abstract

**Significance:**

The multimodality imaging system has become a powerful tool for *in-vivo* biomedical research. However, a conventional multimodality system generally employs two independent detectors, which is costly and bulky. Meanwhile, the geometric cocalibration and image registration between the imaging modalities are also complicated.

**Aim:**

To acquire the multimodality images for small animals with only one visible light sensed single-pixel detector.

**Approach:**

The system is built based on a structured detection Fourier single-pixel imaging architecture. A cesium iodide doped with thallium [CsI(Tl)] scintillator plate is placed behind the sample in x-ray imaging, so the x-ray images can be converted to be visible and sensed with the same single-pixel detector as applied in fluorescence imaging.

**Results:**

The spatial resolution of x-ray imaging was measured to be 1.81 mm, the sensitivity and the imaging depth of fluorescence imaging was evaluated to be ∼1.48  nmol/ml and 4 mm, respectively. *In vivo* multimodality imaging of a C57BL/6 female mouse bearing tumor targeted with mCherry was carried out.

**Conclusions:**

We proposed an x-ray and fluorescence multimodality imaging system for small animals via the structured detection FSI architecture. The system is low cost, with a more compact structure, and free of image registration from different modalities. *In vivo* multimodality imaging results of a mouse bearing tumor demonstrate its capability for small animal research.

## 1 Introduction

*In-vivo* imaging of small animals plays an important role in biomedical research including disease treatment and drug discovery.^1^^–^^3^ Fluorescence imaging is a widely used modality with the advantage of noninvasive detecting, flexible fluorescent probes options, and providing molecular information.^4^ However, a single fluorescence imaging modality lacks precise anatomical information and may lead to incorrect experimental results.^4^ X-ray imaging acquires anatomical information by analyzing the relative attenuation distribution of x-ray passing through the sample. It has been validated in the diagnosis and treatment of skeleton,^5^ stomatology,^6^ and heart diseases.^7^ Combining x-ray imaging with fluorescence imaging to form a multimodality system, we can not only obtain the additional anatomical information but also improve molecular information revealed by fluorescence imaging with much fewer artifacts.

Conventional multimodality imaging systems combining optical and x-ray imaging require two pixelated detectors, charge-coupled device, or complementary metal–oxide-semiconductor in the visible range and flat-panel detector in x-ray range.^4^^,^^8^^,^^9^ However, pixelated detectors at nonvisible wavebands such as x-rays are costly and complicated to manufacture.^10^ Furthermore, the configuration of this kind of multimodality imaging system employing two pixelated detectors is bulky, especially for those tomography systems, which require the rotation of the whole imaging system.^11^

Single-pixel imaging (SPI) is a computational imaging approach reconstructing two-dimensional (2D) images with only one single-pixel detector. SPI has been proved to be a cheaper alternative for applications in nonvisible wavebands, especially for infrared, terahertz, and x-ray imaging,^12^^–^^18^ as the corresponding single-pixel detectors are low cost and easy to acquire.^10^ Meanwhile, many SPI architectures have demonstrated more compact configurations than conventional systems employing independent detectors for multispectral imaging.^19^^,^^20^ SPIs provide the possibility of lower cost in nonvisible wavebands imaging and a more compact configuration in multiband imaging.

In this work, we propose an approach to achieve x-ray and fluorescence multimodality imaging via the structured detection Fourier single-pixel imaging (FSI) architecture. In x-ray imaging modality, a CsI (Tl) scintillator plate is placed behind the sample, so the x-ray images can be converted to visible and sensed with the same single-pixel detector as applied in fluorescence imaging modality. The system only requires one single-pixel detector to capture multimodality imaging data. The spatial resolution of the x-ray imaging, the detection sensitivity, and the imaging depth of fluorophore in the proposed system are evaluated. We also used a female C57BL/6 mouse bearing tumor targeted with mCherry fluorescence protein to demonstrate the feasibility of our system for small animal research.

## 2 Materials and Methods

### 2.1 Experimental Setup

**Fig. 1:**
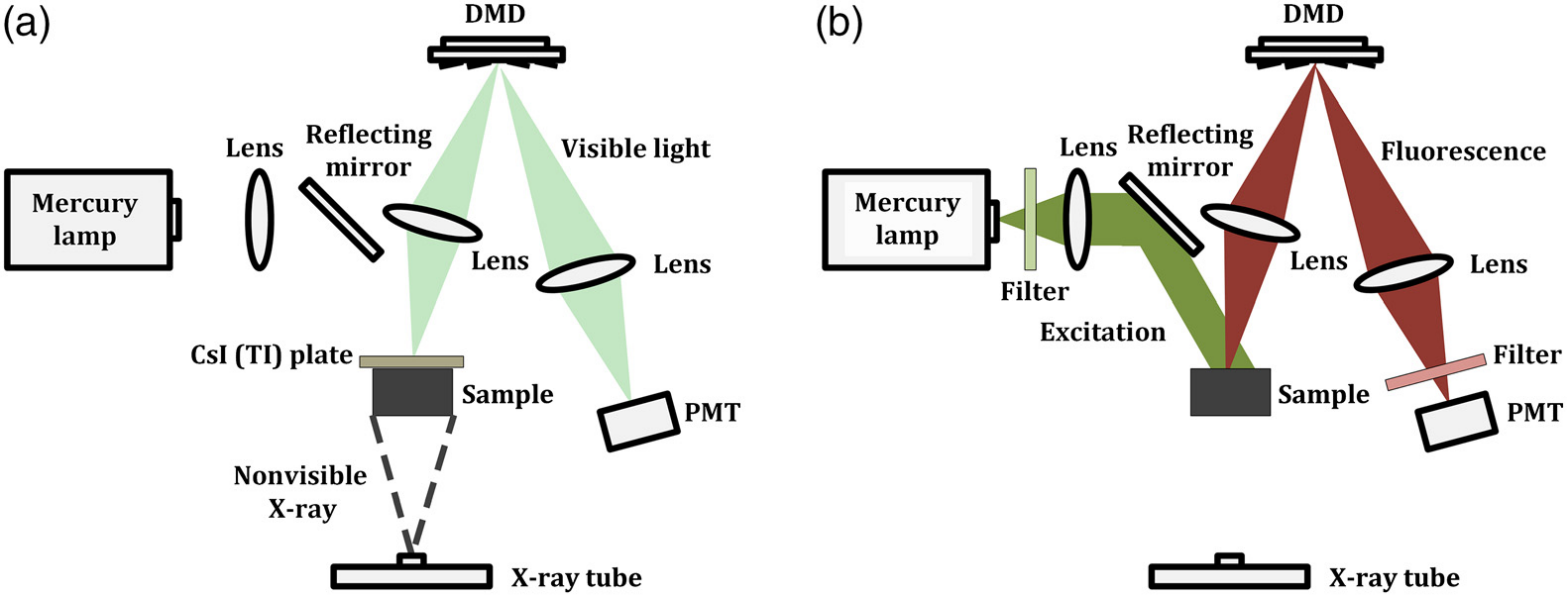
X-ray and fluorescence multimodality FSI system. (a) X-ray imaging configuration. (b) Fluorescence imaging configuration.

The schematic diagram of the proposed x-ray and fluorescence imaging system is shown in Figs. 1(a) and 1(b). The two imaging modalities share the same optical path. An x-ray tube (Oxford Instruments Ultrabright, 50 kV, 0.8 mA) and a mercury lamp (Lumen Dynamics X-Cite Exacte, 200 W, 340 to 675 nm) are employed as the corresponding excitation source. The x-ray beam is conical, and the excitation light emitted from the mercury lamp is collimated. The sample is positioned horizontally and imaged on a digital micromirror device (DMD, X-digit XD-ED01N, 0.7 in., 768×1024 micromirrors, pixel size $13.6\times 13.6\;{\mu m}^{2}$) with a lens (Thorlabs AC254-035-A, focal length 35 mm). Structured detection is achieved by the micromirrors in DMD, turning on and off separately about the diagonal axis. And the modulated image is collected by a photomultipliers tube (Thorlabs PMM02, 300 to 800 nm) as the single-pixel detector.

In x-ray imaging, the spatial modulation of x-ray is hard to realize due to the lack of effective modulator.^21^ Here, we place a CsI (Tl) scintillator plate (Hamamatsu Photonics J8734, effective area of $48\times \;48\;{\mathrm{mm}}^{2}$, 150-μm-thick CsI scintillator) behind the sample. And the nonvisible x-ray containing the anatomical information can be converted to visible light and detected by the photomultipliers tube. The structured detection of x-ray image with the FSI architecture using DMD can be realized. To maximize the imaging field of view, the sample is placed along the diagonal of the scintillator plate, and the DMD chip is tilted for binary modulation. In this way, we can acquire the nonvisible x-ray and visible fluorescence images with only one visible light sensed single-pixel detector.

### 2.2 Data Acquisition and Image Reconstruction

**Fig. 2:**
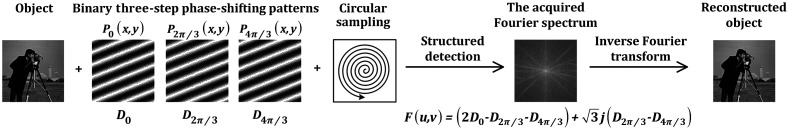
Principle of structured detection with three-step phase-shifting FSI architecture.

The principle of structured detection with three-step phase-shifting FSI architecture is shown in Fig. 2. Instead of sampling in the spatial domain, FSI acquires the Fourier spectrum of the object image.^22^^,^^23^ For a certain Fourier coefficient F(u,v) of an M×N pixels image I, three Fourier basis patterns $P_{Ø}(x,y,u,v)$ with initial phase Ø (Ø=0,2π/3,4π/3 rad) are needed for structured detection:

$$P_{Ø}(x,y,u,v)=\frac{1}{2}+\frac{1}{2}\cos(2\pi \frac{u}{M}x+2\pi \frac{v}{N}y+Ø),$$ (1)

u,v, and x,y separately represent the 2D Cartesian coordinates in the Fourier and space domain. The patterns are binarized by Floyd–Steinberg dithering for fast FSI, and the corresponding responses $D_{Ø}$ can be shown as

$$D_{Ø}=\overset{M}{\underset{x=1}{\sum }}\overset{N}{\underset{y=1}{\sum }}I(x,y)P_{Ø}(x,y,u,v)+n,$$ (2)

where n represents the noise term. The Fourier coefficient F(u,v) can be obtained with the calculation

$$F(u,v)=(2D_{0}-D_{\;2\pi /3}-D_{\;4\pi /3})+\sqrt{3}j(D_{\;2\pi /3}-D_{4\pi /3}),$$ (3)

and the object image can be reconstructed by the inverse Fourier transform using the acquired Fourier spectrum. Differential detection in FSI reduces the noise term, making the architecture more robust, and the sparsity of natural scenes in Fourier domain also accelerates the imaging process.^22^^,^^24^

### 2.3 Animal Preparation

A C57BL/6 mouse (female, SPF, 4 weeks, 10 g) was subcutaneously inoculated with $5\times 10^{5}$ B16 cells targeted by mCherry fluorescence protein. Once the tumor reached a volume of $100\;{\mathrm{mm}}^{3}$, the mouse was anesthetized, and *in vivo* imaging was carried out using our system. All animal experiments were performed according to the animal experiment guidelines of the Animal Experimentation Ethics Committee of Huazhong University of Science and Technology.

## 3 Results

### 3.1 System Performance Evaluation

To evaluate the performance of the proposed system, we measured the spatial resolution of x-ray imaging and the detection sensitivity as well as the imaging depth of fluorescence imaging. In the following experiments, we reconstruct the image with 96×128  pixels. The pixel size in object space is $0.52\times 0.52\;{\mathrm{mm}}^{2}$, and the sampling ratio is 14% with circular sampling method applied in the Fourier domain. DMD refreshes at 20 Hz, and the acquisition time is 2 min for each image. No postprocessing methods were applied to the following results of our system.

**Fig. 3:**
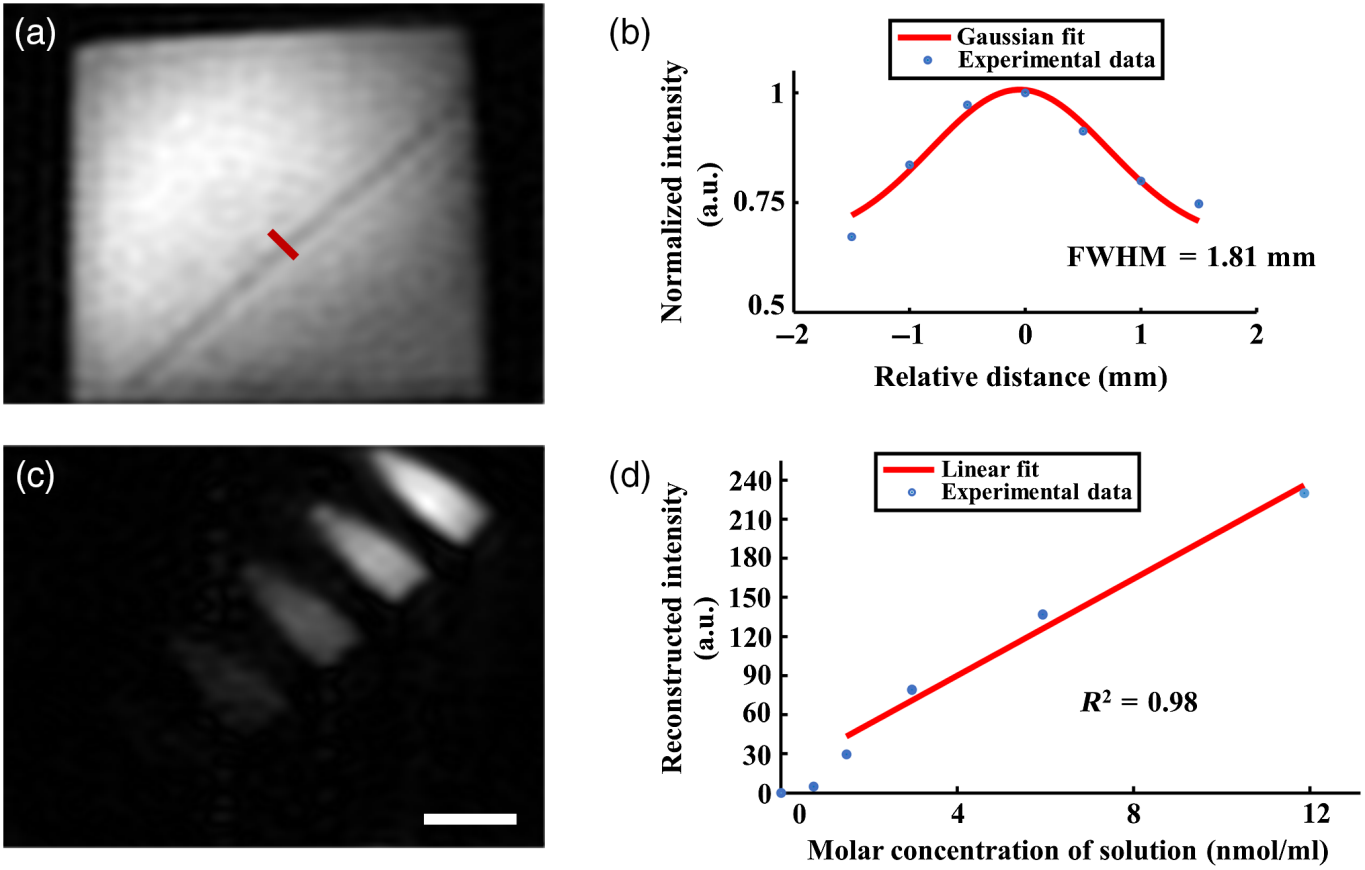
The spatial resolution and fluorescence sensitivity evaluation of the proposed multimodality FSI system. (a) X-ray imaging result of a 0.4-mm-diameter metal wire, 96×128  pixels. (b) Intensity profile with the corresponding Gaussian fit along the line indicated in (a), the full width at half maximum is 1.81 mm. (c) Fluorescence imaging result of five serially diluted rhodamine 6G solutions and pure water, from right to left, 96×128  pixels. (d) Reconstructed intensity of the five rhodamine 6G solutions and pure water with 5×5  pixels averaging. Linear fit to the four detectable Rhodamine 6G solutions’ fluorescence intensity, $R^{2}=0.98$. Scale bars: 10 mm.

As shown in Fig. 3(a), to estimate the spatial resolution of x-ray imaging, a metal wire with diameter of 0.4 mm was imaged. Reconstructed intensity along the line in Fig. 3(a) is shown in Fig. 3(b). Gaussian fitting to the profile of the metal wire indicates the spatial resolution of x-ray imaging is about 1.81 mm.

Then, we evaluated the detection sensitivity of fluorophore of the system in fluorescence modality. 200-μl Rhodamine 6G solution with the serially diluted concentration of 11.86, 5.93, 2.97, 1.48, and 0.74 nmol/ml and pure water as the control group were imaged with the excitation light of 520 to 540 nm. The detected fluorescence ranges from 560 to 580 nm. As shown in Fig. 3(c), the pure water is placed leftmost with the concentration of rhodamine 6G solution reduced from right to left. We calculated the reconstructed intensities of different samples with 5×5  pixels averaging in Fig. 3(c). Linear fitting of the reconstructed fluorescence intensity with $R^{2}=0.98$ suggests that the minimum detectable concentration of rhodamine 6G solution is better than 1.48 nmol/ml.

**Fig. 4:**
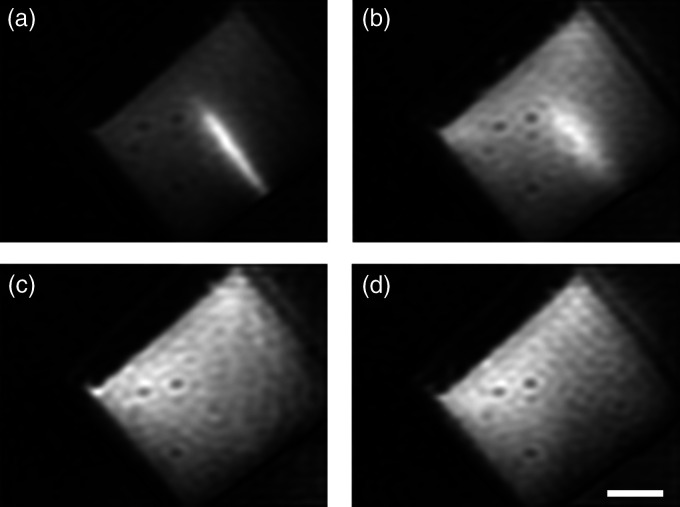
The imaging depth evaluation of the proposed multimodality FSI system using a phantom test. (a)–(d) The imaging results of a glass tube with diameter of 1 mm injected with rhodamine 6G solution and immersed at the depth of 2, 4, 6, and 8 mm in 1 wt% intralipid solution, 96×128  pixels. Scale bars: 10 mm.

Next, to demonstrate the imaging depth of fluorophore in fluorescence modality, the intralipid solution with 1 wt% was chosen as an optical phantom of biological tissue.^25^ The optical property of the phantom can be characterized by the absorption coefficient $\mu_{a}=\;0.03\;{\mathrm{cm}}^{-1}$^26^ and reduced scattering coefficient $\mu_{s}^{\prime }=12.3\;{\mathrm{cm}}^{-1}$.^27^ Rhodamine 6G solution with the concentration of 11.86 nmol/ml was injected to a glass tube with diameter of 1 mm as the fluorescence probe. As shown in Figs. 4(a)–4(d), the glass tube was immersed at the depth of 2, 4, 6, and 8 mm in intralipid solution, respectively, the phantom test was carried out with the excitation light of 520 to 540 nm and detected fluorescence ranges from 560 to 580 nm. In Figs. 4(a) and 4(b), the glass tube can be clearly distinguished from the intralipid solution, so the imaging depth of the system in fluorescence modality can reach 4 mm.

### 3.2 In Vivo Multimodality Imaging of Tumor Bearing Mouse

To further examine the feasibility of the proposed system for small animal research, a C57BL/6 female mouse bearing tumor targeted with mCherry was imaged using our system. A commercial scientific complementary metal-oxide-semiconductor camera (Hamamatsu Photonics ORCA-Flash4.0 V3, 2048×2048  pixels, pixel size: $6.5\times 6.5\;{\mu m}^{2}$) was employed to validate our experimental results.

**Fig. 5:**
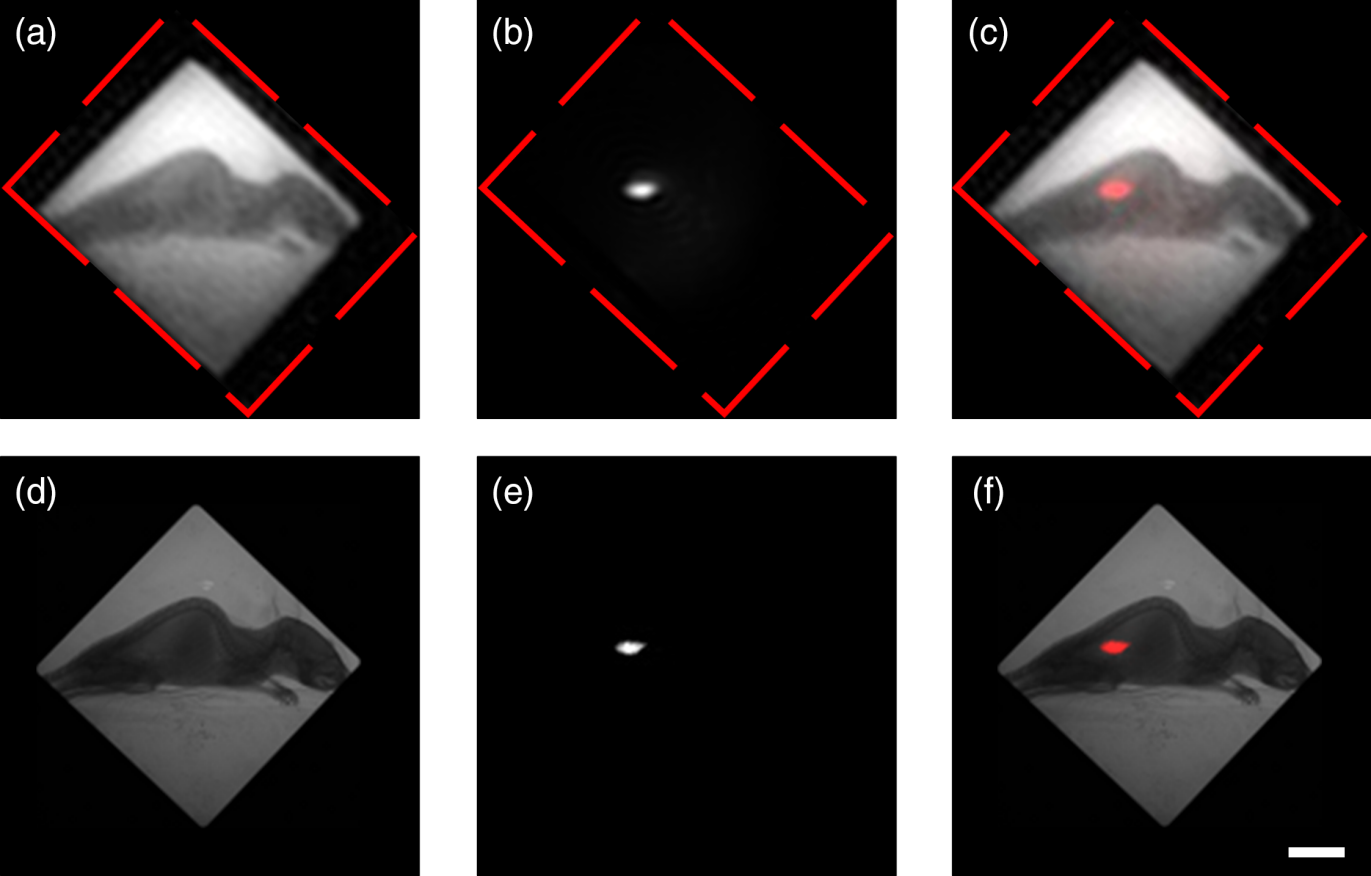
Multimodality imaging results of a C57BL/6 mice bearing tumor targeted with mCherry fluorescence protein using the proposed FSI system (top row, rotated to a specific angle and framed by the red dotted line, 96×128  pixels) and the commercial sCMOS camera (bottom row, 165×165  pixels). (a)–(c) The x-ray, fluorescence, and merged images, respectively. (d) and (e) The corresponding images captured with the sCMOS camera. Scale bars: 10 mm.

In fluorescence imaging modality, the fluorescence of the targeted tumor was excited with light from 560 to 580 nm and detected in the 600 to 620 nm range. The same experimental set up and parameters concerning the reconstructed image of our system were employed as mentioned in Sec. 3.1. The imaging results are shown in Fig. 5. The original FSI results are rotated to a specific angle for better comparison and framed by the red dotted line. Figures 5(a)–5(c) show the x-ray image, fluorescence image, and merged image, respectively. And Figs. 5(d) and 5(e) show the corresponding images captured with the sCMOS camera, respectively. When we acquired the images with an sCMOS camera, the results were resized to 165×165  pixels with pixel size of $0.52\times 0.52\;{\mathrm{mm}}^{2}$ in the object space. And the exposure time of the sCMOS camera is 5 s and 10 s for x-ray imaging and fluorescence imaging, respectively.

As shown in Fig. 5(a), the x-ray imaging is able to provide the necessary anatomical information, including oral cavity, skeleton, and soft tissue. Figure 5(b) shows the molecular information of the small animal, which indicated the location of the tumor cell. And the merged image from the two modalities will provide the anatomical and molecular information simultaneously. The images acquired from the sCMOS camera confirm the results from our system.

## 4 Discussion and Conclusion

We proposed a novel 2D x-ray digital radiography combined with the planar reflective fluorescence multimodality imaging system for small animals via the FSI architecture. Compared with a conventional multimodality imaging system employing two pixelated detectors, only one visible light sensed single-pixel detector is used in our system. The nonvisible x-ray containing the anatomical information is converted to be visible light by a CsI (Tl) scintillator plate. Therefore, x-rays and fluorescence images can be reconstructed by the structured detection FSI architecture using DMD. The spatial resolution of x-ray imaging is measured to be 1.81 mm, and the detection sensitivity as well as the imaging depth of rhodamine 6G is approaching picomolar and 4 mm. A C57BL/6 female mouse bearing tumor targeted with mCherry is used to demonstrate the feasibility of our system for small animal studies.

Except for the low cost and compact structure, we have shown that free of image registration is also an advantage of our system, given by the detection with the same detector. It is worth noting that the imaging speed and imaging resolution of the two modalities in our system are worse than the conventional ones. Recently, the SPI architectures employing the deep learning method have been demonstrated to achieve fast imaging with high resolution. Meanwhile, traditional methods of optimizing the sampling strategy also accelerate the imaging procedure. With the approaches mentioned above, we may improve the imaging speed and imaging resolution of our system and expect better performance in further three-dimensional imaging, time domain imaging, and multispectral imaging.

## Code, Data, and Materials Availability

All relevant code, data, and materials are available from the authors upon reasonable request. Correspondence and requests should be addressed to the corresponding author.
